# Seasonal variation of viral infections between the eastern honey bee (*Apis cerana*) and the western honey bee (*Apis mellifera*)

**DOI:** 10.1002/mbo3.1162

**Published:** 2021-02-04

**Authors:** Gongwen Chen, Yuqi Wu, Jie Deng, Zhengsheng Wen, Shuai Wang, Yanping Chen, Fuliang Hu, Huoqing Zheng

**Affiliations:** ^1^ College of Animal Sciences Zhejiang University Hangzhou China; ^2^ Bee Research Laboratory USDA‐ARS Beltsville MD USA

**Keywords:** *Apis cerana*, *Apis mellifera*, prevalence, variation, viruses

## Abstract

It is a widespread practice in China to keep colonies of both the western honey bee, *Apis mellifera*, and the eastern honey bee, *Apis cerana*, in close proximity. However, this practice increases opportunities for spillover of parasites and pathogens between the two host bee species, impacting spatial and temporal patterns in the occurrence and prevalence of the viruses that adversely affect bee health. We conducted a 1‐year large‐scale survey to assess the current status of viral infection in both *A. mellifera* and *A. cerana* in China. Our study focused on multiple aspects of viral infections in honey bees, including infection rate, viral load, seasonal variation, regional variation, and phylogenetic relationships of the viruses within the same species found in this study and other parts of the world. The survey showed that the black queen cell virus (BQCV), deformed wing virus (DWV), Israeli acute paralysis virus (IAPV), and sacbrood virus (SBV) were common in both *A. mellifera* and *A. cerana*, and infection dynamics of BQCV, DWV, and SBV between bee species or seasons were significantly different. DWV was the most common virus in *A. mellifera*, and its infection rate and load in *A. mellifera* were higher than those in *A. cerana*, which reflects the high susceptibility of *A. mellifera* to *Varroa destructor* infestation. The infection rate and viral load of SBV were higher in *A. cerana* than in *A. mellifera*, indicating that SBV poses a greater threat to *A. cerana* than to *A. mellifera*. Our results also suggested that there was no geographical variation in viral dynamics in *A. mellifera* and *A. cerana*. Phylogenetic analyses of BQCV, DWV, IAPV, and SBV suggested the cross‐regional and cross‐species spread of these viruses. This study provides important insights into the complex relationships between viruses and their hosts in different seasons and regions, which will be important for developing effective disease management strategies to improve bee health.

## Graphical abstract

We conducted a 1‐year large‐scale survey to provide an update on the current status of the virus infection in both *Apis mellifera* and *Apis cerana* in China. Our study focused on multiple aspects of virus infection in honey bees including infection rate, viral load, seasonal variation, and phylogenetic relationship of the viruses with the same bee species found in other parts of the world.

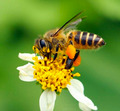

## 1 INTRODUCTION

Honey bees (*Apis* spp.) are the most important insect pollinators of food crops worldwide, playing an irreplaceable role in food security, ecological sustainability, and biodiversity (Gallai et al., 2009; Klein et al., 2007; Potts et al., 2016). Populations of *Apis mellifera*, the most widely distributed and managed honey bee, have suffered great losses in many countries in recent decades (Brodschneider et al., 2016, 2018; Potts et al., 2010; VanEngelsdorp & Meixner, 2010). Of all stress factors, viruses are frequently associated with worldwide bee health decline, especially with regard to winter mortality and colony losses (Chantawannakul et al., 2016; Desai et al., 2016; Ratnieks & Carreck, 2010; Steinhauer et al., 2018).

Currently, more than 30 viruses have been identified in honey bee populations (Beaurepaire et al., 2020). The most common and widely spread viruses include the acute bee paralysis virus (ABPV), black queen cell virus (BQCV), chronic bee paralysis virus (CBPV), deformed wing virus (DWV), Israeli acute paralysis virus (IAPV), Kashmir bee virus (KBV), and sacbrood virus (SBV). ABPV, KBV, and IAPV are part of a complex of closely related viruses from the family *Dicistroviridae* with relatively high virulence (Miranda et al., 2010). ABPV and IAPV have been reported to result in rapidly progressing paralysis and death of honey bees after experimental inoculation (Bailey et al., 1963; Maori et al., 2007). KBV can cause great damage to a colony when the levels of the parasitic mite *Varroa destructor* are high (Chen et al., 2004). BQCV is also a member of the family *Dicistroviridae* and primarily harms queen larvae and pupae, causing the rapid death of queen brood (Bailey & Woods, 1977). CBPV, a currently unclassified virus, causes chronic paralysis in adult bees, leading to the death of adult workers and spreading to other colony members (Bailey & Woods, 1974). DWV and SBV are members of the family *Iflaviridae*. The typical disease symptom caused by DWV is a wing deformity. The association between DWV and the parasitic mite *V. destructor* has caused significant damage to colonies (Allen & Ball, 1996; Di Prisco et al., 2016; Martin et al., 2010; Miranda & Genersch, 2010) and has recently been a primary cause of bee colony mortality worldwide (Schroeder & Martin, 2014). SBV mainly infects brood and results in larval death (Nguyen & Le, 2013). It is lethal to *Apis cerana* but less detrimental to *A. mellifera* (Gong et al., 2016).

*A. mellifera* and *A. cerana* are bee species used in the beekeeping industry in China at a national level and are often kept close to each other. While large‐scale epidemiological investigations of honey bee viruses in China have been conducted in previous studies (Ai et al., 2012; Diao et al., 2019; Li et al., 2012; Yang et al., 2013), a broader comparative analysis of viral infections in both host species simultaneously would yield critical insights into the relationship between viruses and their hosts. Furthermore, most viruses infecting honey bees are RNA viruses that have extremely high mutation rates, which could have important epidemiological consequences. We therefore conducted a 1‐year large‐scale survey to assess the current status of viral infections in *A. mellifera* and *A. cerana* in China. This study focused on multiple aspects of viral infections in honey bees, including infection rate, viral load, seasonal and regional variation, and phylogenetic relationships with the same viruses in these bee species found in other parts of the world. We anticipate that this study will provide important insights into the evolutionary pattern and risk associated with the epidemic of viral disease in honey bees.

## 2 MATERIALS AND METHODS

### 2.1 Sample collection for virus detection

**FIGURE 1:**
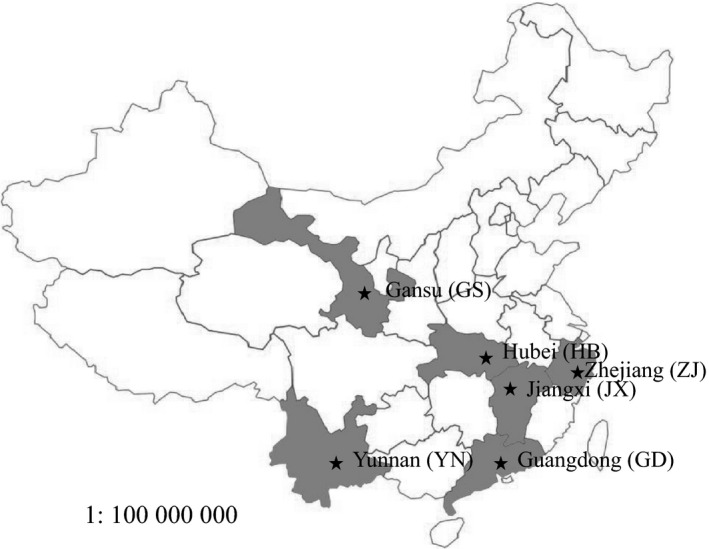
Geographical locations where honey bee samples were collected

Adult workers were sampled from 244 *A. mellifera* and 238 *A. cerana* colonies distributed over 45 apiaries in six provinces (Gansu, Hubei, Zhejiang, Jiangxi, Guangdong, and Yunnan) in China (Table 1; Figure 1). In each province, *A. mellifera* and *A. cerana* colonies were sampled in the same or nearby cities. Sample collections were conducted in autumn (October) and winter (December) of 2017 and spring (April) and summer (July) of 2018. All colonies used in this study were assessed for colony health by local beekeepers. No clear disease symptoms were observed in brood and adult workers used in the study.

**TABLE 1:** Synopsis of sample collection

Source of samples (provinces)	Honey bee species	Number of samples			
		Spring	Summer	Autumn	Winter
Gansu (GS)	*A. cerana*	9	9	9	0
	*A. mellifera*	9	9	9	0
Hubei (HB)	*A. cerana*	9	0	9	3
	*A. mellifera*	9	0	9	8
Zhejiang (ZJ)	*A. cerana*	23	20	20	25
	*A. mellifera*	20	25	20	30
Jiangxi (JX)	*A. cerana*	9	9	15	8
	*A. mellifera*	9	9	9	9
Guangdong (GD)	*A. cerana*	9	6	9	9
	*A. mellifera*	9	6	9	9
Yunnan (YN)	*A. cerana*	4	6	9	9
	*A. mellifera*	9	9	3	6

### 2.2 RNA isolation and cDNA synthesis

Thirty workers (Pirk et al., 2015) from each colony were crushed to a fine powder in liquid nitrogen and used for RNA isolation using the RNApure Total RNA Kit (Aidlab Biotechnologies Co. Ltd.), according to the manufacturer's protocol. cDNA synthesis was conducted using 800 ng RNA and ReverTra Ace qPCR RT Master Mix (Toyobo), according to the manufacturer's instructions.

### 2.3 qRT–PCR assays for viral load quantification

Informed by our pilot study and previous epidemiological surveys in China (Ai et al., 2012; Diao et al., 2019; Li et al., 2012; Yañez et al., 2015; Yang et al., 2013), we focused on the five most common bee viruses (BQCV, CBPV, DWV‐A, IAPV, and SBV). qRT–PCR was performed using 1 μl cDNA as the template in 10 μl reactions, using THUNDERBIRD SYBR qPCR Mix (Toyobo) with a StepOne Plus Real‐Time PCR System (Applied Biosystems). The thermal profile of the PCR program consisted of 1 min incubation at 95°C and 40 cycles of 95°C for 15 s and 60°C for 1 min. A melt curve analysis was used to confirm the specificity of the products. Each PCR amplification included a negative control where 1 μl of RNase‐free water was used instead of template cDNA. The primers used for qRT–PCR are shown in Table 2.

**TABLE 2:** Primer sets used for quantitative detection of BQCV, CBPV, DWV, IAPV, and SBV

Virus	Primer sequence (5′−3′)	Position in the complete genome	GenBank accession number	Reference
BQCV	GGAGTCGCAGAGTTCCAAAT	7954–7973	MT096521.1	Lin (2017)
	GTGGGAGGTGAAGTGGCTAT	8075–8059		
CBPV	GGCACCTCAAGATCGTCCAAGTTAC	348–372	KY937971.1	This study
	ACGGAGATGGTGACCTGGTATGG	487–465		
DWV (DWV‐A)	CGTGGTGTAGTAAGCGTCGT	6676–6694	KX373899.2	This study
	TCATCCGTAGAAAGCCGAGT	6795–6776		
IAPV	TCGCTGAAGGCATGTATTTC	486–505	MG599488.1	Lin (2017)
	ATTACCACTGCTCCGACACA	617–598		
SBV	AACGTCCACTACACCGAAATGTC	468–490	MN082651.1	Blanchard et al. (2014)
	ACACTGCGCGTCTAACATTCC	537–517		

Viral loads were quantified using absolute quantification methods. The linear standard curve equation for each virus was based on three linear standard curves obtained through six ten‐fold dilutions of known amounts of plasmids (pMD^®^18‐T Vector, TaKaRa) containing cloned viral target sequences (Wu et al., 2017). A linear standard curve was used for each qRT–PCR run.

### 2.4 RT‐PCR amplification and sequencing

For each sample, 1 μl cDNA was used for PCR amplification using KOD FX (TOYOBO) according to the manufacturer's instructions. PCR products were electrophoresed in 2% agarose gels, purified, and sequenced by a commercial company (Sangon Biotch). The sequence specificity of each virus was checked by sequencing analysis using the NCBI BLAST service. The primer pairs used for RT‐PCR in this study are shown in Table 3.

**TABLE 3:** Primer sets used for PCR amplification of BQCV, DWV, IAPV, and SBV

Virus	Primer sequence (5′–3′)	Position in the complete genome	GenBank accession number	Reference
BQCV	GTGGCGGAGATGTATGCGCTTTATC	7791–7815	MN565034.1	Yang et al. (2013)
	CTGACTCTACACACGGTTCGATTAG	8434–8410		
DWV (DWV‐A)	GTCGTGCAGCTCGATAGGAT	8960–8941	KX373899.2	Tentcheva et al. (2004)
	TTTGCAAGATGCTGTATGTGG	8566–8586		
IAPV	AGACACCAATCACGGACCTCAC	8955–8976	MG599488.1	Maori et al. (2007)
	AGATTTGTCTGTCTCCCAGTGCACAT	9429–9404		
SBV	GGATGAAAGGAAATTACCAG	7747–7766	KY273489.1	Tentcheva et al. (2004)
	CCACTAGGTGATCCACACT	8172–8154		

### 2.5 Phylogenetic analysis

The sequences of BQCV, DWV, IAPV, and SBV isolates obtained from this study were individually aligned using Clustal W (Thompson et al., 1997) with other representative homologous sequences retrieved from GenBank. The phylogenetic trees were constructed using MEGA7 software with the Maximum Likelihood method based on the Tamura 3‐parameter model (Tamura, 1992) and a bootstrap value of 1000 replicates.

### 2.6 Data analysis

The arcsine transformation was conducted to convert the infection rate (%). The log copy numbers were used for the statistical analysis of viral loads. The results of Levene's test for homogeneity of variance showed that the viral infection rate and load data did not meet the conditions for the multivariate analysis of variance (MANOVA), so we performed a paired sample *t‐*test to compare the viral infection rate and load between species (*p* < 0.05), seasons (*p* = 0.008 after Bonferroni correction [Yekutieli & Benjamini, 2001]), and provinces (*p* = 0.003 after Bonferroni correction). Multiple infections of viruses between *A. mellifera* and *A. cerana* were compared using a chi‐square test, and values of *p* < 0.05 were considered statistically significant.

## 3 RESULTS

### 3.1 Viral infection dynamics in different provinces

**FIGURE 2:**
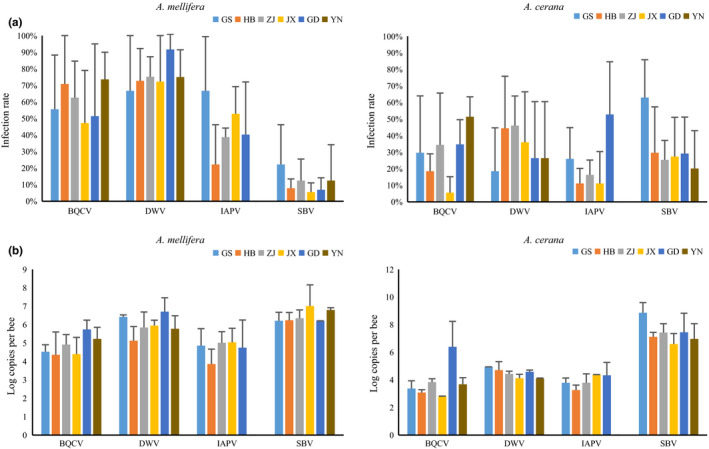
Infection rates (a) and viral loads (b) of BQCV, DWV, IAPV, and SBV in *A. mellifera* and *A. cerana* at the six provinces. Error bars represent standard deviations

BQCV, DWV, and SBV could be detected in *A. mellifera* and *A. cerana* in each province, while IAPV was absent in samples from Yunnan. CBPV was detected only in *A. mellifera* samples from Zhejiang and Yunnan in winter. There was no statistical difference in the infection status of BQCV, DWV, IAPV, and SBV between the provinces for viral infection dynamics in *A. mellifera* and *A. cerana* (Figure 2).

### 3.2 Virus infection dynamics in different seasons

**FIGURE 3:**
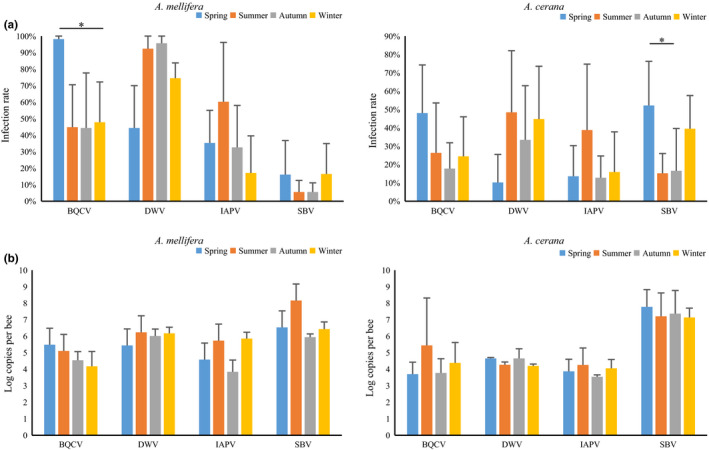
Comparison of infection rates (a) and viral loads (b) of BQCV, DWV, IAPV, and SBV in *A. mellifera* and *A. cerana* among four seasons. * represents significance level at *p* < 0.00833. Error bars represent standard deviations

Seasonally, viral infection rates of BQCV in *A. mellifera* peaked in spring and then fell (*p* = 0.007, spring vs. winter), and infection rates of SBV in *A. cerana* also peaked in spring (*p* = 0.007, spring vs. summer; *p* = 0.003, spring vs. autumn; Figure 3a). DWV infection in *A. mellifera* showed a different seasonal pattern, with a strong increase during autumn (*p* = 0.002, spring vs. autumn). There was no significant difference in the viral load between seasons for the four viruses (Figure 3b). IAPV was the only virus with no significant difference in infection dynamics between seasons for either *A. mellifera* or *A. cerana*.

### 3.3 Viral infection dynamics in different bee species

**FIGURE 4:**
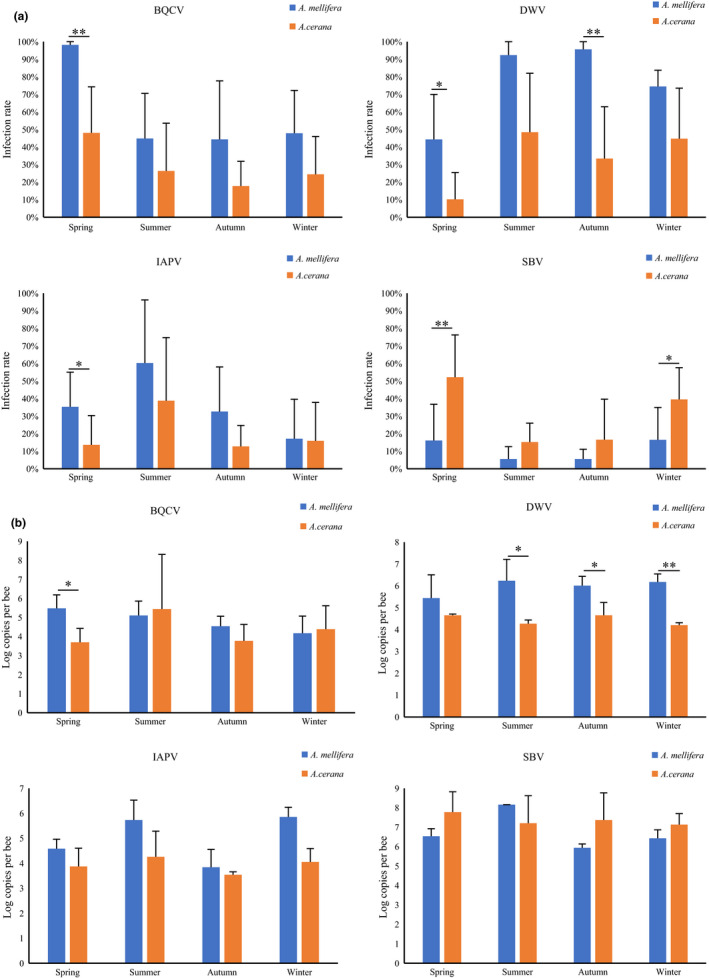
Comparison of infection rates (a) and viral loads (b) of BQCV, DWV, IAPV, and SBV between *A. mellifera* and *A. cerana* at four seasons. * represents significance level at *p* < 0.05; ** represents significance level at *p* < 0.01. Error bars represent standard deviations

There were statistical differences in the infection dynamics of BQCV, DWV, IAPV, and SBV between *A. mellifera* and *A. cerana*. The infection rates of BQCV, DWV, and IAPV in *A. mellifera* were significantly higher than those in *A. cerana* in spring (*p* = 0.003; *p* = 0.019; *p* = 0.025) and of DWV in autumn (*p* = 0.008), while the infection rates of SBV in *A. mellifera* in spring and winter (*p* = 0.018; *p* = 0.037) were significantly lower than those in *A. cerana* (Figure 4a). Besides, loads of BQCV in spring (*p* = 0.016) and of DWV in summer (*p* = 0.042), autumn (*p* = 0.045), and winter (*p* = 0.001) in *A. mellifera* were higher than those in *A. cerana* (Figure 4b).

**FIGURE 5:**
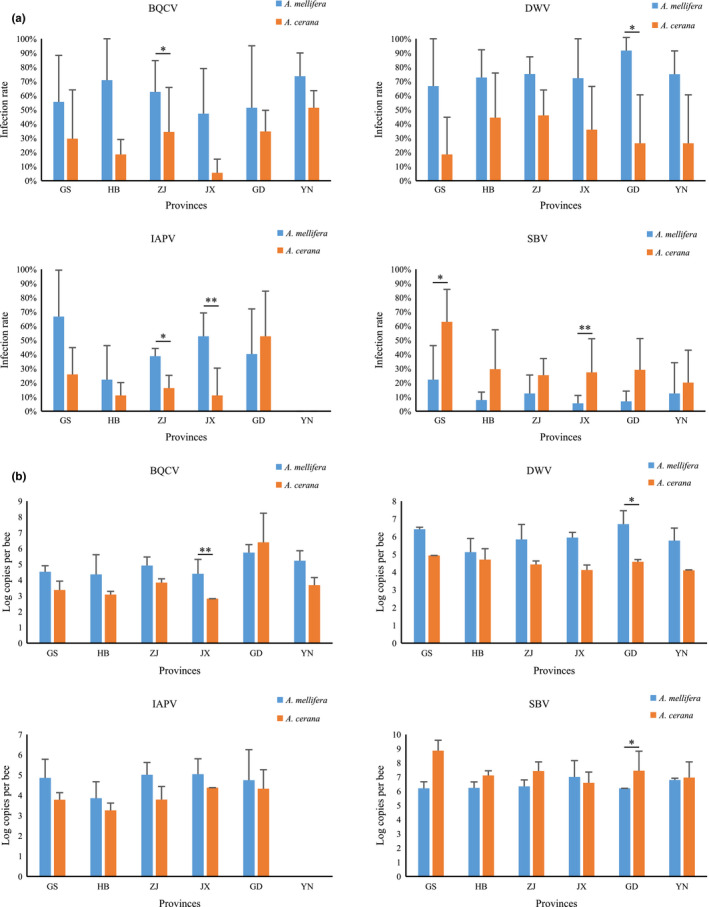
Comparison of infection rates (a) and viral loads (b) of BQCV, DWV, IAPV, and SBV between *A. mellifera* and *A. cerana* at the six provinces. * represents significance level at *p* < 0.05; ** represents significance level at *p* < 0.01. Error bars represent standard deviations

Significant differences in the comparison of viral prevalence between *A. mellifera* and *A. cerana* in the same province were found in BQCV in Zhejiang (*p* = 0.020), DWV in Guangdong (*p* = 0.023), IAPV in Zhejiang (*p* = 0.038), Jiangxi (*p* = 0.003), SBV in Gansu (*p* = 0.026), and Jiangxi (*p* = 0.004; Figure 5a). The comparison of infection loads between *A. mellifera* and *A. cerana* in the same province indicated that significant differences occurred in the viral loads of BQCV in Jiangxi (*p* = 0.003), DWV (*p* = 0.025), and SBV (*p* = 0.010) in Guangdong (Figure 5b).

**FIGURE 6:**
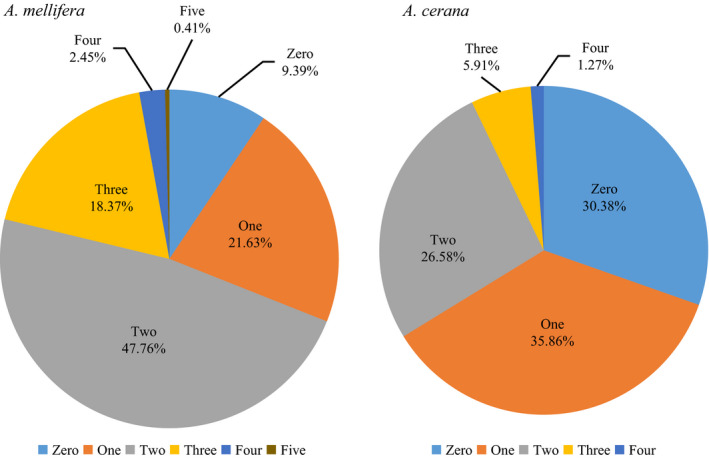
Multiple viral infections in *A. mellifera* and *A. cerana*

The proportion of *A. mellifera* colonies that were infested with none, one, two, three, four, or five viruses were 9.39%, 21.63%, 47.76%, 18.37%, 2.45%, and 0.41%, respectively. For *A. cerana*, the proportion was 30.38%, 35.86%, 26.58%, 5.91%, 1.27%, and 0.00%, respectively (Figure 6). Thus, the proportion of *A. cerana* colonies infected with no or one virus was significantly higher (χ^2^ = 35.78, *p* < 0.01; χ^2^ = 12.61, *p* < 0.01), while the proportion of *A. mellifera* colonies infected with two or three viruses was significantly higher (χ^2^ = 23.08, *p* < 0.01; χ^2^ = 17.41, *p* < 0.01).

### 3.4 Phylogenetic relationship

**FIGURE 7:**
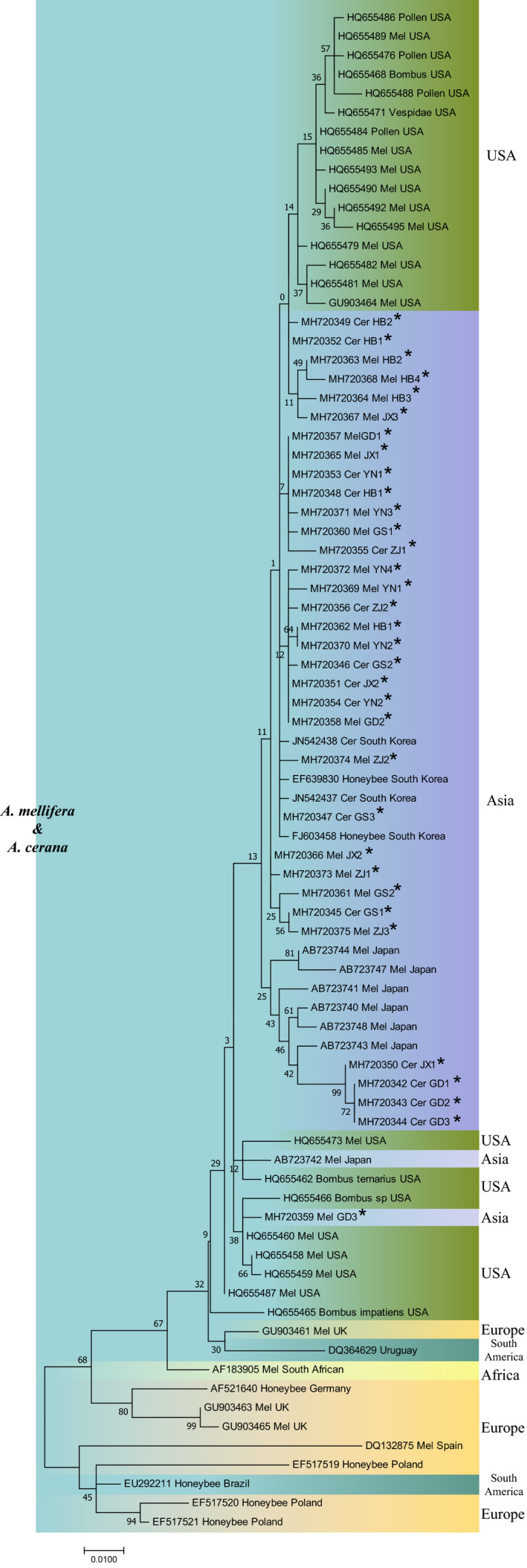
Phylogenetic tree of BQCV. Notes: Isolates identified in this study are flagged by asterisks. Isolates are marked with different colors according to their countries or continents of origin (right) and host (left). Isolates are annotated with respect to GenBank accession number, virus‐host, and geographical origin of isolates. Mel: *A. mellifera*; Cer: *A. cerana*

**FIGURE 8:**
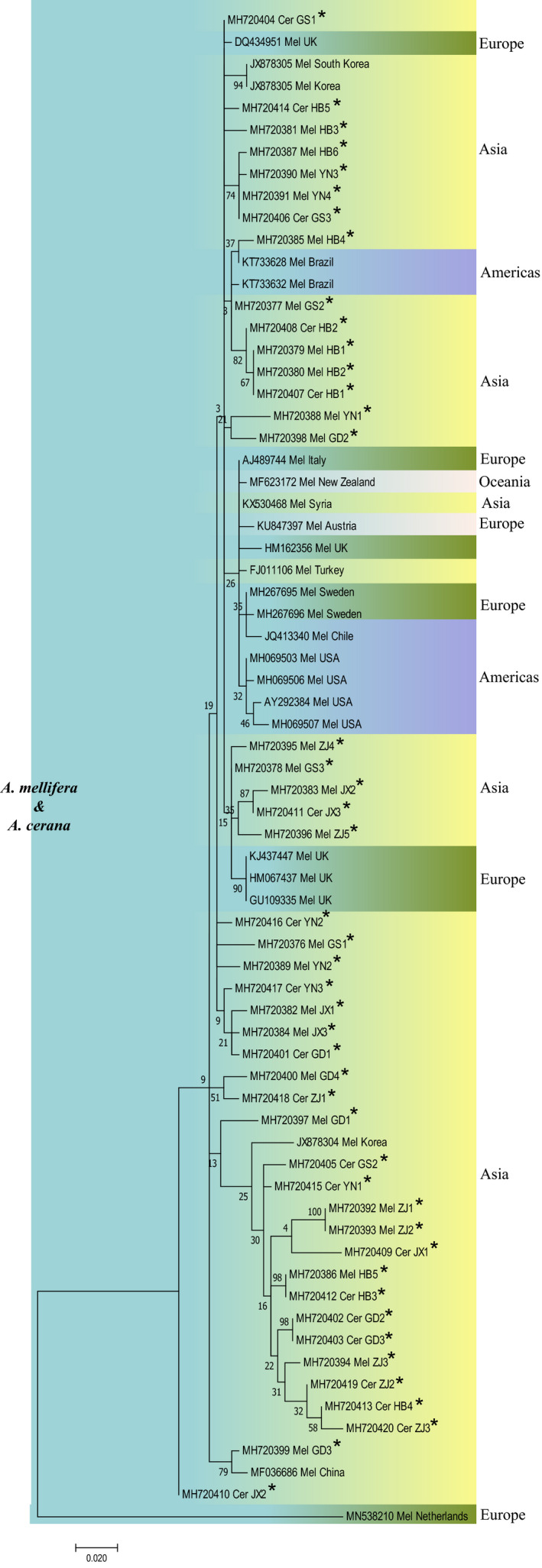
Phylogenetic tree of DWV. See the legend in Figure 7

**FIGURE 9:**
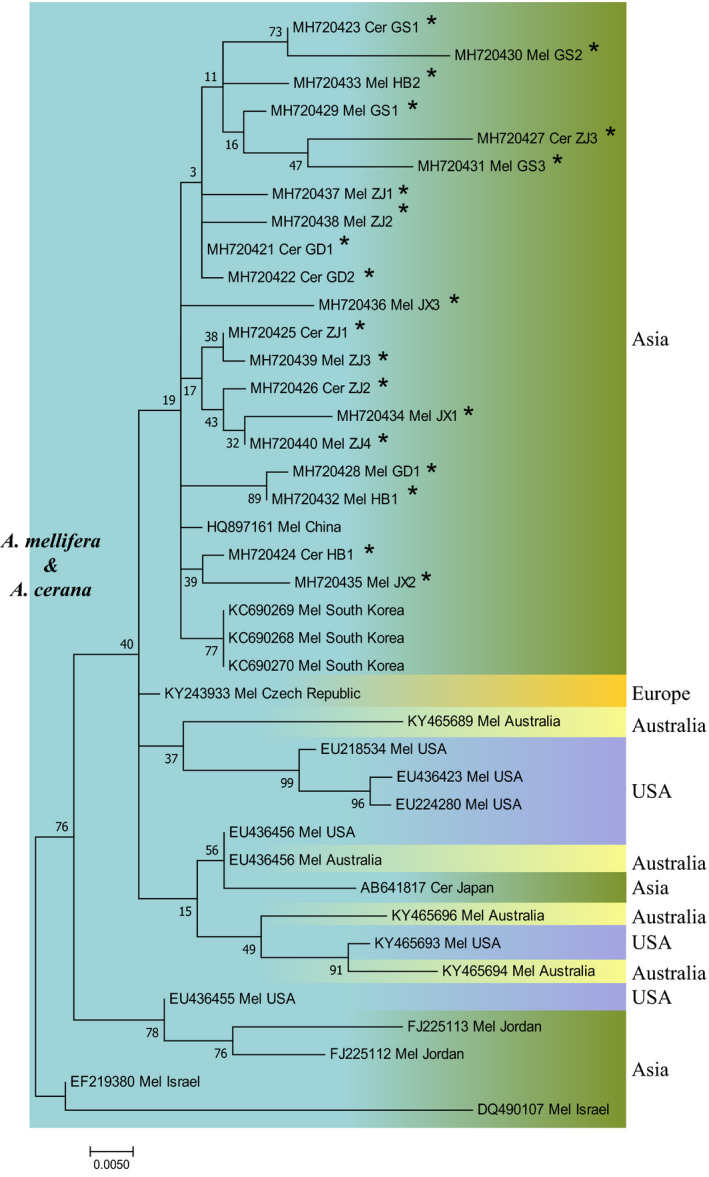
Phylogenetic tree of IAPV. See the legend in Figure 7

All BQCV, IAPV, and DWV isolates from *A. mellifera* and *A. cerana* in different regions of China were phylogenetically clustered together and did not form distinct clades based on the species of the host. All of the BQCV isolates from Asia and the United States were clustered into the same clade, which was isolated from another clade mainly composed of European isolates (Figure 7). DWV isolates from China were clustered into several clades with isolates from South Korea, Brazil, and the UK (Figure 8). The IAPV isolates from China formed a common clade with isolates from South Korea, Australia, Japan, the United States, and the Czech Republic (Figure 9).

**FIGURE 10:**
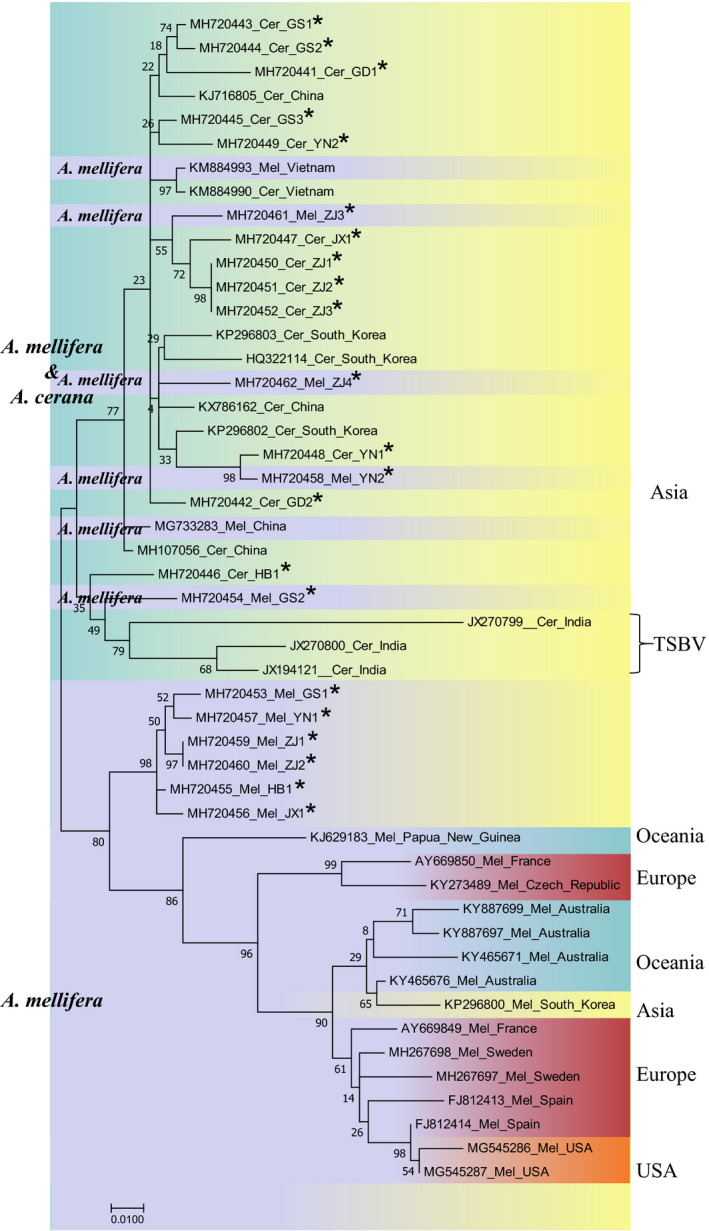
Phylogenetic tree of SBV. See the legend in Figure 7

The SBV phylogenetic tree consisted of two branches. One branch consisted of Asian SBV isolates from both *A. mellifera* and *A. cerana*, which further split into two subbranches. The first one was dominated by *A. cerana* SBV isolates but also contained six *A. mellifera* SBV isolates from Vietnam and China. Among the six isolates, four were identified in this study, which accounted for 14.28% (4/28) of the total sequenced *A. mellifera* SBV isolates. Another subbranch consisted of six *A. mellifera* SBV isolates from China. The second branch consisted of SBV isolates from *A. mellifera* from Oceania, Europe, Asia, and North America (Figure 10).

## 4 DISCUSSION

Our work provided the first seasonal data revealing common virus prevalence in both *A. mellifera* and *A. cerana* occurring at the same time in China. In this survey, BQCV, CBPV, DWV, IAPV, and SBV were all detected, with the most common viruses being BQCV and DWV, which was consistent with investigations conducted in 2009 and 2012 (Ai et al., 2012; Ding & Shi, 2015). Simultaneous multiple infections of honey bee viruses have been reported (Chen et al., 2005; Chen, Zhao, et al., 2004), and our results also showed that multiple infections were common in *A. mellifera* and *A. cerana* in China. The high infection rate and load of DWV in *A. mellifera*, which has been regarded as potentially responsible for *A. mellifera* colony losses (Nordström et al., 1999), suggested that it is an important factor affecting the health of *A. mellifera* in China. Moreover, the impact of the widespread prevalence of BQCV on *A. mellifera* in China is noteworthy because of its high pathogenicity to queen larvae (Anderson, 1993) and the large royal jelly production industry in China (Zheng et al., 2018). SBV was first identified in *A. mellifera* in the United States in 1913 and was subsequently found in *A. cerana* in Thailand (Thai sacbrood viruses, TSBV) and China (Chinese sacbrood viruses, CSBV; Allen & Ball, 1996; Bailey et al., 1982; Zhi & Chou, 2008). SBV infection in *A. mellifera* rarely leads to colony death, whereas the infection of *A. cerana* SBV (including TSBV and CSBV) is fatal to *A. cerana* (Blanchard et al., 2014; Kshirsagar & Phadke, 1985; Zhi & Chou, 2008), which has caused serious losses to *A. cerana* colonies in many Asian countries, including Vietnam, Thailand, India, China, and South Korea (Choe et al., 2012; Liu et al., 2010; Nguyen & Le, 2013; Rana et al., 1986). Our results suggested that SBV is common in *A. cerana* in China, which was corroborated by previous surveys (Ai et al., 2012; Ding & Shi, 2015; Yañez et al., 2015); therefore, the adverse effects of SBV on *A. cerana* deserve special attention. In this study, the overall infection rate of IAPV in *A. cerana* reached 18.57%, which was higher than that in previous reports (7% in the survey by Ai et al. and 0%−12.2% in the survey by Ding et al.; Ai et al., 2012; Yang et al., 2013), suggesting that *A. cerana* in China may potentially be facing an increasing threat from this virus. Also, we found that the infection dynamics of BQCV, DWV, IAPV, and SBV between *A. mellifera* and *A. cerana* in certain seasons were significantly different, which suggested that the infection pattern and dynamics of the same virus in *A. mellifera* and *A. cerana* vary.

Our study also allowed us to compare the prevalence and titers of the five common honey bee viruses in *A. mellifera* and *A. cerana* colonies throughout six provinces of China. The results indicated that there was no regional variation in the infection status of BQCV, DWV, IAPV, and SBV among different provinces in *A. mellifera* and *A. cerana*. This can be explained by migratory beekeeping, which facilitated the transfer of bee viruses between different provinces (Zheng et al., 2018).

Previous studies have reported seasonal changes in bee viral infections in *A. mellifera* (Natsopoulou et al., 2017; Runckel et al., 2011; Tentcheva et al., 2004). Our results also showed that seasonality impacted the prevalence of BQCV and DWV in *A. mellifera* and that of SBV in *A. cerana*. The infection rate of BQCV in *A. mellifera* was found to be high in spring, then decreased in summer, and remained stable in summer, autumn, and winter. However, a national investigation conducted in France showed that the infection rate of BQCV in summer was significantly higher than that in spring and autumn, and an investigation conducted in southwest Germany showed that BQCV frequencies and titers in *A. mellifera* were significantly lower in spring than in autumn (Natsopoulou et al., 2017; Tentcheva et al., 2004), which was different from our results. The discrepancies between these studies might be due to climate differences among the different countries or the genetic differences in the honey bees or viruses. The infection rate of DWV in *A. mellifera* increased during summer, which agreed with previous surveys conducted in France and China (Diao et al., 2019; Tentcheva et al., 2004) and could be related to the increase in *V. destructor* density from spring to summer (Gloria et al., 2017). However, due to a lack of data on the parasitism rate of *V. destructor* in this study, a more comprehensive epidemiological investigation is warranted.

Phylogenetic analysis of BQCV, DWV, and IAPV did not show distinct patterns of phylogenetic clustering by host species, suggesting cross‐species transmission of these viruses between *A. mellifera* and *A. cerana* (Yañez et al., 2015; Yang et al., 2013). The lack of geographical separation of BQCV, DWV, IAPV, and SBV isolates from these six provinces implied that they freely spread across provinces. Previous studies have reported the cross‐species transmission of *A. cerana* SBV from *A. cerana* to *A. mellifera* (Gong et al., 2016). In this study, we also noticed that four SBV isolates from *A. mellifera* were clustered with *A. cerana* SBV, which accounted for 14.28% (4/28) of the total sequenced *A. mellifera* SBV isolates in our study. This result indicated the gradual weakening of the obstacles to the inter‐species transmission of SBV between *A. mellifera* and *A. cerana*. Although the pathogenicity of *A. cerana* SBV seems to be weak in *A. mellifera* (Gong et al., 2016), it is important to conduct further studies on its substantive effect and to determine how SBV interacts with *A. mellifera* and *A. cerana*, which will improve our understanding of viral pathogenic mechanisms in honey bees and developing new and innovative treatment approaches.

## 5 CONCLUSION

This study provides an overview of the infection dynamics of five common viruses in *A. mellifera* and *A. cerana* over different seasons and across regions in China. The results of this study provide important insights into the complex relationships between viruses and their hosts and are relevant for developing effective disease management strategies to improve bee health. Nevertheless, considering the limitations of the study due to the short sampling period (1 year), more comprehensive epidemiological surveys in a larger area and over longer sampling periods for both honey bee species in the future will provide broader insights.

## CONFLICT OF INTEREST

None declared.

## ETHICS STATEMENT

None required.

## AUTHOR CONTRIBUTION

**Gongwen Chen:** Investigation (lead); Methodology (lead); Writing‐original draft (lead); Writing‐review & editing (lead). **Yuqi Wu:** Methodology (equal); Writing‐review & editing (equal). **Jie Deng:** Investigation (equal). **Zhengsheng Wen:** Investigation (equal). **Shuai Wang:** Methodology (equal). **Yanping Chen:** Methodology (equal); Writing‐review & editing (equal). **Fu‐Liang Hu:** Conceptualization (equal). **Huoqing Zheng:** Conceptualization (equal); Project administration (lead); Resources (lead); Supervision (lead); Writing‐review & editing (equal).

## Data Availability

A total of 121 nucleotide sequences of BQCV, DWV, IAPV, and SBV obtained in this study are available in the GenBank database: https://www.ncbi.nlm.nih.gov/nuccore. The accession numbers are as follows: MH720342 to MH720375 (BQCV), MH720376 to MH720420 (DWV), MH720421 to MH720440 (IAPV), and MH720441 to MH720462 (SBV).
